# Cost-Effectiveness of Thyroid Nodule Molecular Testing in Manitoba

**DOI:** 10.1177/19160216261479282

**Published:** 2026-09-18

**Authors:** Erika Lee, Rana Chakrabarti, Norbert Viallet, Alok Pathak, Suhail Sayed, Paul Kerr, Ciaran Lane

**Affiliations:** 1 Department of Otolaryngology – Head and Neck Surgery, University of Manitoba College of Medicine, Winnipeg, MB, Canada; 2 Department of Pathology, University of Manitoba College of Medicine, Winnipeg, MB, Canada; 3 Head and Neck Surgical Oncology, Department of Otolaryngology – Head and Neck Surgery, University of Manitoba College of Medicine, Winnipeg, MB, Canada; 4 Head and Neck Surgical Oncology, Department of General Surgery, University of Manitoba College of Medicine, Winnipeg, MB, Canada

**Keywords:** thyroid nodule, molecular testing, cost-effective

## Abstract

**Objective:**

To determine the cost-effectiveness of molecular testing for indeterminate (Bethesda III/IV) thyroid nodules in a real-world, single-payer healthcare system.

**Design:**

This retrospective population-based cohort study analyzed fine-needle aspiration biopsy (FNAB) and lobectomy data for thyroid nodules in Manitoba, Canada, during 2023. Microcosting was performed to capture surgical, surveillance, molecular testing, and complication costs in 2023 Canadian dollars. Six molecular tests (Afirma, ThyroSeqV3, ThyroidPrint, ThyroSPEC, ThyGeNEXT/ThyraMIR, and mir-THYpe) were evaluated using site-specific malignancy prevalence (21%) and published test statistics.

**Setting:**

The study was performed within a publicly funded healthcare system.

**Participants:**

Participants were 18 years and above having undergone FNAB in Manitoba.

**Intervention or Exposures:**

Three scenarios were identified Current Care, Molecular Testing, and Reference Standard (diagnostic lobectomy) pathways. The total cost per year were compared among scenarios.

**Main Outcome Measures:**

Cost-utility analyses compared Current Care, Molecular Testing, and Reference Standard pathways. Quality-adjusted life-years (QALYs) and incremental cost-effectiveness ratios (ICERs) were calculated. A $50 000/QALY willingness-to-pay (WTP) threshold was used to interpret ICERs.

**Results:**

Among 1486 FNABs performed, 413 were indeterminate nodules, and 149 patients underwent diagnostic lobectomy. The cost of lobectomy was $7542.69 per patient. All molecular testing strategies improved net QALYs and demonstrated ICERs ranging from $9791.03 to $58 749.94 per QALY compared to Current Care. All tests apart from Afirma were cost-effective at the WTP threshold relative to Current Care. Compared to the Reference Standard, all molecular testing strategies were dominant.

**Conclusions:**

For the investigated Manitoba scenario, molecular testing for indeterminate thyroid nodules can be a cost-effective strategy, possibly reducing unnecessary surgeries while preserving patient outcomes.

**Relevance:**

This study contributes to the body of evidence suggesting molecular testing is cost-effective and has potential to influence political decisions in health care spending.

## Graphical abstract

**Graphical Abstract:**
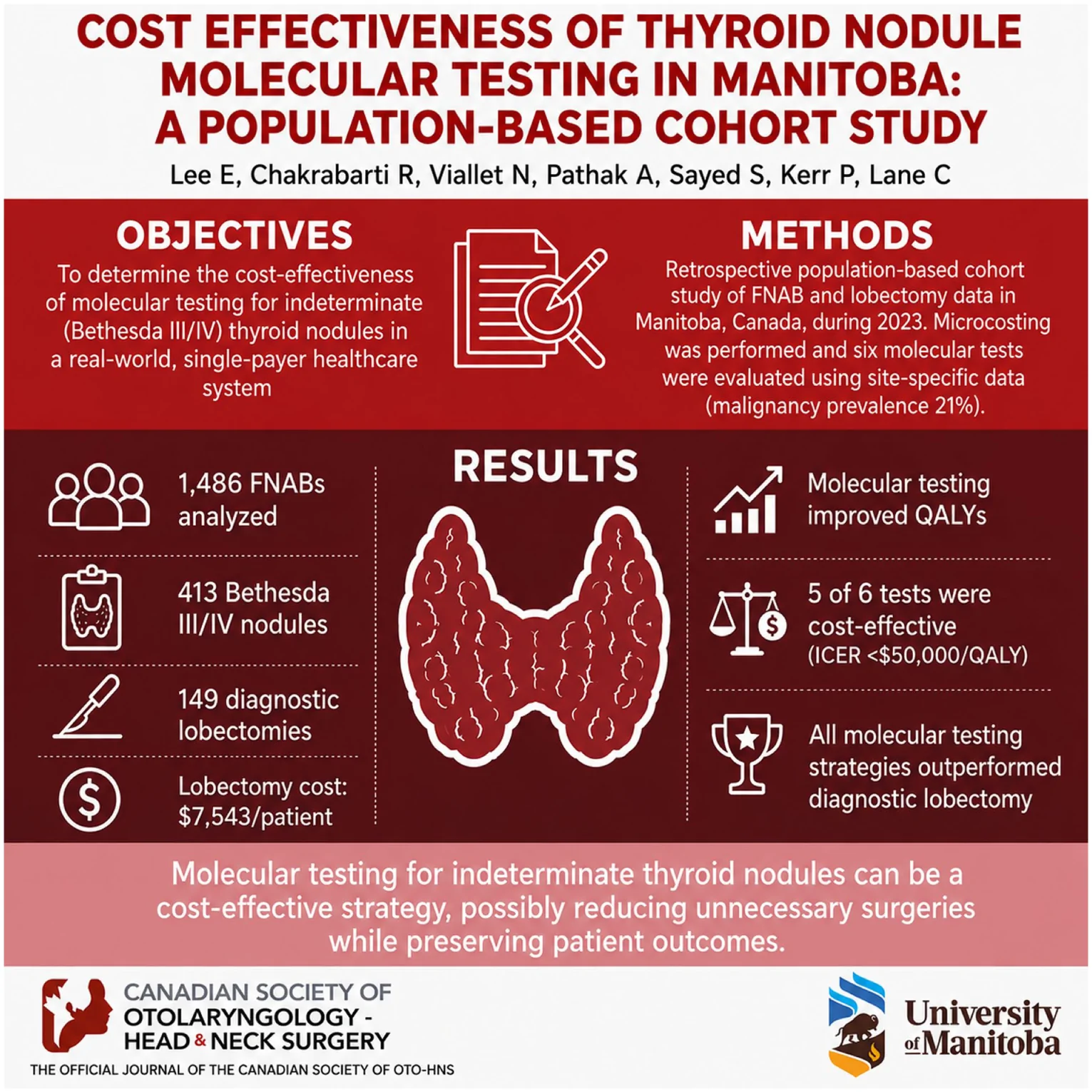
This is a visual representation of the abstract.

## Key Message

The addition of thyroid molecular testing in the investigated Manitoba setting has the potential for cost savings in a publicly funded setting.

## Background

Thyroid nodules are common growths occurring in up to 50% of adults over the age of 60 and are commonly discovered incidentally on imaging. The vast majority are benign, but 5% represent malignancy.^1^ Approximately 140 new thyroid cancer cases are diagnosed annually in Manitoba.^2^ Well-differentiated thyroid cancers can be treated successfully with surgery; however, delays in management can result in transformation of differentiated cancers into highly aggressive forms such as anaplastic thyroid cancer.^1^

Thyroid nodule evaluation begins with an ultrasound, followed by fine-needle aspiration biopsy (FNAB) of nodules meeting the Thyroid Imaging, Reporting, and Data System (TI-RADS) criteria.^3^ Cytology from FNAB enables the stratification of patients with the Bethesda System for Reporting Thyroid Cytopathology into those who are at high, intermediate (or indeterminate), and low risk for cancer.^4^ The management of stratified nodules is guided by the American Thyroid Association (ATA) guidelines.^5^

The reference diagnostic standard for thyroid cancer is to perform diagnostic lobectomy for indeterminate nodules, however the risk of malignancy in indeterminate nodules is on average 22% to 30% and unnecessary lobectomy can result in high cost to the healthcare system and increased patient anxiety.^4^ Thus patients may opt to undergo repeat FNAB and surveillance ultrasounds.

Thyroid molecular testing has been added to the Bethesda system to further stratify malignancy risk.^4^ Canadian provincial governments have started to address the addition of molecular testing to the care pathway. Currently Newfoundland, Southern Alberta, and Quebec have molecular testing coverage. Governmental and academic bodies have performed cost analyses with varying degrees of cost-effectiveness.

The limitations of previous cost testing are that probability estimates are used and propose that all indeterminate nodules receive lobectomy.^6,7^ The advantage of this study is its representation of molecular testing in the real world with a resource restricted environment.

The outcome of this study is to determine the cost-effectiveness of molecular testing within Manitoba. We speculate that the implementation molecular thyroid testing into the care pathway will reduce unnecessary surgeries and will be cost-effective within this healthcare system. The results of this study may inform policy change.

## Materials and Methods

### Study Design and Problem

This population-based retrospective cohort study was conducted in Manitoba, Canada, to evaluate the cost-effectiveness of molecular testing for indeterminate thyroid nodules (Bethesda III and IV) within a publicly funded healthcare system. The study was approved by the University of Manitoba Research Ethics Board (REB#HS26812) and reported in accordance with Canada’s Drug Agency (CDA).

### Literature Review

A literature review was performed to identify commercially available molecular tests for indeterminate thyroid nodules. PubMed, MEDLINE, and EMBASE were searched from January 1, 2015 to present using the terms “indeterminate thyroid nodule molecular test.” Studies reporting validated diagnostic performance metrics, including sensitivity, specificity, positive predictive value (PPV), and negative predictive value (NPV), were included. Six molecular tests met inclusion criteria: Afirma, ThyroSeq v3, ThyroidPrint, ThyroSPEC, ThyGeNEXT/ThyraMIR, and mir-THYpe. Tests with lower diagnostic performance (miRInform and RosettaGX) were excluded. Reported NPVs ranged from 91% to 97%, supporting their role as rule-out tests.

Afirma was developed in San Francisco, CA, USA and uses next-generation RNA sequencing.^8,9^ ThyroSeqV3 from Sonic Healthcare is a genomic classifier using next-generation sequencing of DNA and RNA.^10^ ThyroidPrint is a quantitative PCR-based 10-gene classifier made in Santiago, Chile.^11^ ThyroSPEC is a DNA and RNA mass spectrometry test based in Calgary, Canada.^12^

ThyGeNEXT/ThyraMIR uses a genotyping panel combined with a microRNA-based assay. Samples are tested through ThyGeNEXT and if a typical oncogenic mutation (such as BRAF V600E) is not found, samples then undergo ThyraMIR. Mir-THYpe is a microRNA test from Brazil created in 2018.^13^ The cost, sensitivities, specificities, NPV, and PPV of each test can be found in Table 1. The NPV of these tests range from 91% to 97%, allowing for use as a rule out test.^14^

**Table 1.** Comparison of the Performance and Cost of Available Thyroid Molecular Tests.

Thyroid Molecular Test	Test type	Sensitivity	Specificity	Negative predictive value	Positive predictive value	Cost per test (CAD)^a^
Afirma (San Francisco, CA, USA)^b^	RNA Next Gen Seq	91%	68%	96%	65%	$4316
ThyroSeqV3 (New York, NY, USA)^c^	DNA and RNA Next Gen Seq	94%	82%	97%	66%	$4785
ThyroidPrint (Santiago, Chile)^d^	Quantitative PCR mRNA expression of 10 genes	91%	88%	95%	78%	$2000
ThyroSPEC (Calgary, AB, Canada)^e^	DNA and RNA mass spectrometry	67%-74%	57%-79%	76%-91%	46%-65%	$800
ThyGeNEXT/ThyraMIR (Pittsburgh, PA, USA)^f^	DNA Next Gen Seq + miRNA	95%	90%	97%	75%	$2708
Mir-THYpe (São Paulo, Brazil)^g^	miRNA	89%	82%	95%	66%	$2300

a Email communication with company regarding pricing in Canadian Dollars (CAD).

b Patel et al (2018)^8^ and Vardarli et al (2024)^9^ for recent numbers.

c Steward et al (2019)^10^.

d Olmos et al.^11^

e Stewardson et al.^12^

f Lupo et al.^14^

g Santos et al.^13^

### Economic Evaluation and Time Horizon

A cost-utility analysis was conducted from the perspective of the Canadian publicly funded healthcare system. Costs were reported in 2023 Canadian dollars. A 1 year time horizon was used to reflect diagnostic phase of care from identification of indeterminate nodule to surgery or surveillance. Lifetime quality-adjusted life year (QALY) losses were also incorporated.

### Target Population

The modeled population reflects adult patients with indeterminate thyroid nodules (Bethesda III/IV). All FNAB results from 2023 were reviewed. Patients with Bethesda III/IV cytology were included and duplicate biopsies in the same patient were excluded. Surgical pathology records were used to correlate cytology with final histopathology where available. The pooled malignancy prevalence of Bethesda III/IV was 21%.

### Comparators

Three management strategies were evaluated: (1) Current Care, defined as real-world management with either surveillance or diagnostic hemithyroidectomy based on clinician-patient decision-making; (2) Molecular Testing, in which all indeterminate nodules underwent reflex molecular testing, with management determined by test results; and (3) a Reference Standard in which all patients underwent diagnostic hemithyroidectomy without molecular testing. The Reference Standard was included as a theoretical comparator.

### Model Structure

A decision-tree model was developed to simulate patient pathways over a 1 year period using 2023 Manitoba cohort data (Figures 1 and 2). Patients in the Current Care arm were categorized into surgical or surveillance pathways based on observed practice patterns. In the Molecular Testing arm, the number of true positives, false positives, true negatives, and false negatives for each test was estimated using a malignancy prevalence of 21% and published test performance characteristics (Table 1 and Supplemental Table 4a-f). Positive test results were assumed to lead to diagnostic hemithyroidectomy, while negative results resulted in discharge without further surveillance. In the Reference Standard arm, patients underwent reflexive diagnostic hemithyroidectomy. We acknowledge that the Reference Standard is a dominated strategy compared to Current Care. However, its inclusion provides a theoretical benchmark and maintains consistency with prior literature.^5^ The primary conclusions of this study are drawn from comparisons with Current Care.

**Figure 1.**
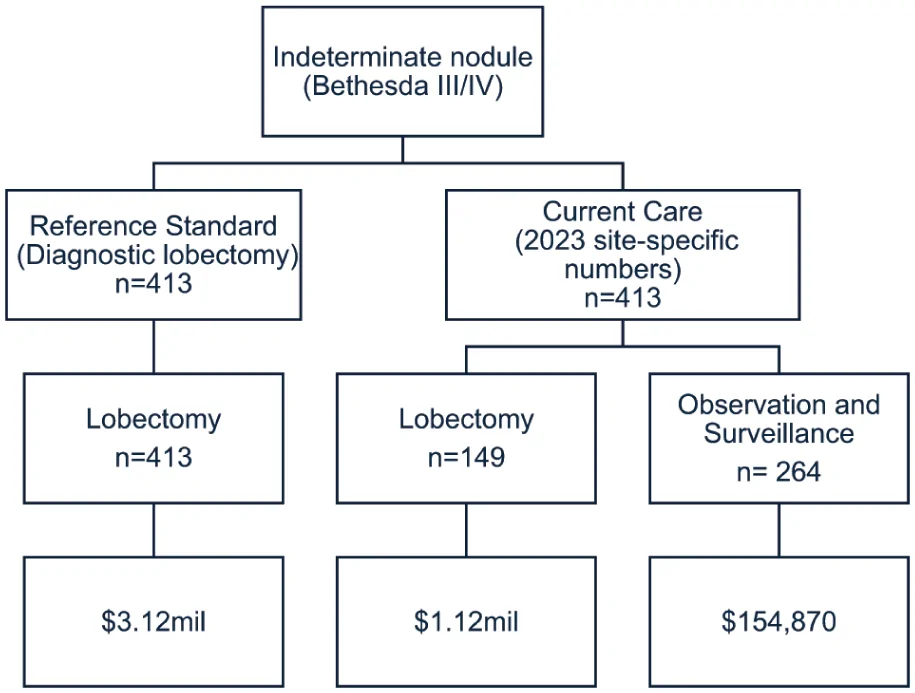
Total yearly cost in Reference Standard and Current Care scenarios.

**Figure 2.**
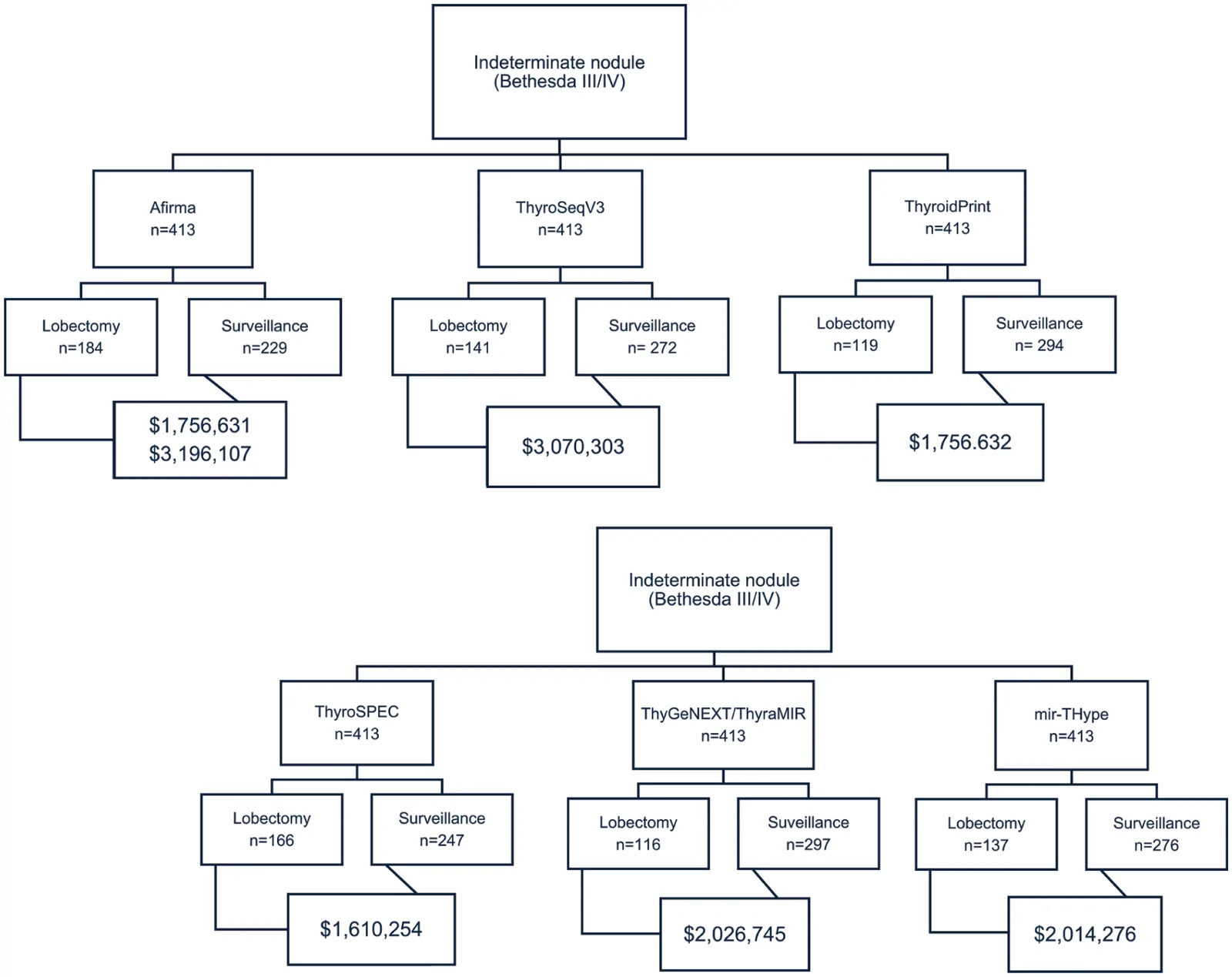
Total yearly cost in 6 Molecular Test scenarios (ThyroidPrint, ThyroSPEC, ThyGeNEXT/ThyraMIR, Afirma, ThyroSeqv3, mir-THYpe).

### Costs

Costs included direct medical expenditures relevant to the public payer, including operating room costs, FNAB processing and interpretation, physician visits, laboratory investigations, and ultrasound imaging (Supplemental Tables 1-3). Complication costs were incorporated using published rates for hematoma (1.5%), wound infection (1%), and recurrent laryngeal nerve injury (1%), with associated resource use including emergency or outpatient visits and laryngology consultation. Hypoparathyroidism was not included, as the model was limited to hemithyroidectomy. Long-term medication costs were excluded to reflect the healthcare payer perspective. Post-hemithyroidectomy hypothyroidism requiring thyroid hormone supplementation occurs in approximately 10% to 30% of patients. However, because our analysis focused on healthcare system costs relevant to provincial budgeting, patient-level medication costs were not included. The costs were weighted by the average incidence of complication in the literature.^5,14,15^ Unit costs were derived from Manitoba Shared Health databases, provincial physician billing codes, pathology service communications, and local institutional data.

### Measurement of Health

Health outcomes were expressed in QALYs. Utility values of 0.98 and 0.99 were applied to post-hemithyroidectomy and surveillance health states, respectively, based on published estimates.^6,17^ A mean age at diagnosis of 53 years and life expectancy of 82 years were assumed.^18^ For the single mortality event observed in the Current Care arm, QALY loss was calculated over the patient’s remaining life expectancy (49 years) to capture the full impact of this preventable death. This approach is consistent with standard practice in cost-effectiveness analyses where mortality is a relevant outcome, as truncating QALY loss to a 1 year horizon would substantially underestimate the true burden of missed cancers. A 2% risk of progression to aggressive disease was applied to false-negative cases. The Reference Standard strategy assumed no QALY loss from missed malignancy.

### Model Analysis and Scenario Analysis

Total costs and QALYs were calculated for each strategy, and incremental cost-effectiveness ratios (ICERs) were derived by dividing the difference in costs by the difference in QALYs between strategies. Scenario analyses were performed to assess model robustness, including variation in malignancy prevalence (15%-30%) and surgical costs (±40%). Malignancy prevalence was varied (base case 21%) to reflect the range reported in the literature for Bethesda III and IV nodules.^6,7,19,20^ Probabilistic sensitivity analysis was not performed.

### Discounting

In accordance with CDA standard reporting, costs and outcomes would typically be discounted at 1.5% annually; however, given the 1 year analytic horizon for costs, discounting was not applied to base-case results.

### Model Validation

The model structure and assumptions were informed by real-world Manitoba data and validated through comparison with published literature.

## Results

There were 1486 FNABs performed in 2023. Of these, there were 413 individuals with indeterminate nodules (Bethesda category III and IV). The rest of the 1073 were Bethesda I, II, V, VI, or were repeat biopsies (Table 2).

**Table 2.** Overview Table – Demographics, number of hemithyroidectomies, FNAB, and deaths.

2023 Data	Total	Bethesda III	Bethesda IV
Diagnostic hemithyroidectomy	149	96	53
Malignant	88	58	30
Benign	61	38	23
FNAB			
Total	1486		
Indeterminate (Bethesda III/IV)	413	344	69
Number of deaths due to disease	1	1	0

Abbreviation: FNAB, fine-needle aspiration biopsy.

### Reference Standard

The costs were estimated for 413 patients undergoing diagnostic lobectomy, including surgeon’s billing fee, operation theater cost, surgery unit, pathology, and complications, followed by a follow up clinic visit was estimated at $7542.69 per person. The cost for the Reference Standard pathway in 2023 was $3 115 130.97 (Figure 1). The net QALY gained was calculated to be 143.1 for 413 patients undergoing lobectomy (Table 3).

**Table 3.** Cost-Utility and Cost-Benefit Analysis and ICER Calculation for Molecular Testing.

Molecular test	Cost per year	Current Care				Reference Standard	
		Difference in cost compared with Current Care	Net QALY (QALY gained − QALY lost)	Difference in QALY compared to Current Care	ICER compared to Current Care ($/QALY)	Difference QALY compared to Reference Standard	ICER MT compared to Reference Standard
Afirma	$3 196 107.14	$1 917 376.01	127.71	32.64	$58 749.94	−15.43	5247.93
ThyroSeqV3	$3 070 302.53	$1 791 571.40	141.20	46.13	$38 838.88	−1.94	23 130.71
ThyroidPrint	$1 756 631.59	$477 900.46	139.54	44.47	$10 747.52	−3.60	377 335.37
ThyroSPEC	$1 610 254.28	$331 523.15	128.93	33.86	$9791.03	−14.21	105 929.02
ThyGeNEXT/ThyraMIR	$2 026 744.78	$748 013.65	140.71	45.64	$16 390.61	−2.43	447 963.00
Mir-THYpe	$2 014 276.45	$735 545.32	138.32	43.24	$17 009.80	−4.82	228 208.29
Reference Standard	$3 115 130.97	$1 836 399.84	143.14	48.07	$38 205.53	0.00	N/A
Current Care	$1 278 731.13	N/A	95.07	N/A	N/A	48.51	38 205.53

Abbreviations: MT, molecular test; ICERs, incremental cost-effectiveness ratios; QALY, quality-adjusted life year.

### Current Care

There were 149 diagnostic lobectomies performed in 2023 (Table 2). The resection rate of all intermediate nodules was 36%. A total of 88 specimens returned as positive for malignancy, all of which were well-differentiated thyroid cancers. The malignancy rate was 21% among resected indeterminate nodules in Manitoba. The pooled rate in our cohort reflects the relative proportions of Bethesda III and IV nodules in our population and falls within the wide range of 14% to 55% reported in Canadian centers.^6,19-21^ The Bethesda categories were then analyzed separately. The resection rate for Bethesda III nodules was 27.9% and malignancy rate was 16.9% which falls within the expected range based on Bethesda guidelines. The resection rate for Bethesda IV nodules was 76.8% and malignancy rate was 43.5%, which was higher than average based on Bethesda guidelines^4^ (Table 2). To be consistent with literature, a combined rate was used in the scenario analysis. The cost of diagnostic lobectomy per person in the Current Care pathway was the same as the Reference Standard ($7542.69).

There were 264 patients who underwent surveillance was totaled at $154 870.32 ($586.63 per patient). The mean cost of Current Care was $3096.20 per person. In total, the cost for Current Care was $1 278 731.13 (Figure 1).

The QALY gained was calculated at 144.1. One patient with initial indeterminate cytology (Atypia of Undetermined Significance, AUS) in the surveillance arm developed anaplastic thyroid cancer and passed away at age 33. The time interval between the initial FNA and diagnosis of stage IVC anaplastic thyroid cancer was several months. This case likely represents either a false negative of the initial biopsy or aggressive transformation of a differentiated thyroid cancer during the surveillance period. While anaplastic thyroid cancer is rare, this observed case reflects a real, albeit uncommon, risk of surveillance strategies at our institution. The QALY lost from this death from aggressive thyroid cancer was 49. The net QALY of Current Care is 95.1.

### Molecular Testing

Overall the total cost of all 6 molecular testing pathways were more expensive compared to Current Care but less expensive compared to the Reference Standard (Figure 2 and Table 3). The ICERs for Molecular Testing compared to Current Care were $10 747.52/QALY gained for ThyroidPrint, $9791.03/QALY gained for ThyroSPEC, $16 062.50/QALY gained for ThyGeNEXT/ThyraMIR, $58 749.94/QALY gained for Afirma, $38 838.88 /QALY gained for ThyroSeqv3, and $17 009.80/QALY gained for mir-THYpe. All ICERs calculated for Molecular Tests are below the $50 000 willingness-to-pay (WTP) threshold, except Afirma. The ICER for Current Care compared to the Reference Standard was −$38 205.53.

Compared to the Reference Standard, ICERs for Molecular Testing subgroups were negative since the Reference Standard is significantly more expensive. A summary of the results comparing Molecular Testing to Current Care and Reference Standard can be found in Table 3. With increasing malignancy prevalence, molecular testing strategies except Afirma were more expensive but below the threshold (Tables 4 and 5). When surgical costs are increased, compared to Current Care all tests were below the WTP threshold apart from Afirma (Supplemental Tables 5 and 6).

**Table 4.** Recalculation of ICER With Current Care QALY Calculation at 15% Prevalence.

Molecular test	Cost per year	Current Care				Reference Standard	
		Difference in cost compared with Current Care	Net QALY (QALY gained − QALY lost)	Difference in QALY compared to Current Care	ICER compared to Current Care ($/QALY)	Difference QALY compared to Reference Standard	ICER MT compared to Reference Standard
Afirma	$3 084 653.09	$1 805 921.96	132.40	37.33	$48 378.18	−10.74	2838.56
ThyroSeqV3	$2 921 697.13	$1 642 966.00	141.85	46.78	$35 121.85	−1.29	150 261.22
ThyroidPrint	$1 608 026.19	$329 295.06	140.77	45.70	$7206.08	−2.37	636 040.10
ThyroSPEC	$1 535 951.58	$257 220.45	134.77	39.70	$6479.88	−8.37	188 646.64
ThyGeNEXT/ThyraMIR	$1 863 278.84	$584 547.71	141.95	46.87	$12 470.48	−1.19	1 050 361.96
Mir-THYpe	$1 873 101.32	$594 370.19	140.12	45.05	$13 193.67	−3.02	411 716.99
Reference Standard	$3 115 130.97	$1 836 399.84	143.14	48.07	$38 205.53	0.00	N/A
Current Care	$1 278 731.13	N/A	95.07	N/A	N/A	48.51	38 205.53

Abbreviations: MT, molecular test; ICERs, incremental cost-effectiveness ratios; QALY, quality-adjusted life year.

**Table 5.** Recalculation of ICER With Current Care QALY Calculation at 30% Prevalence.

Molecular test	Cost per year	Current Care				Reference Standard	
		Difference in cost compared with Current Care	Net QALY (QALY gained − QALY lost)	Difference in QALY compared to Current Care	ICER compared to Current Care ($/QALY)	Difference QALY compared to Reference Standard	ICER MT compared to Reference Standard
Afirma	$3 352 142.81	$2 073 411.68	132.28	37.20	$55 733.95	−10.86	21 815.48
ThyroSeqV3	$3 278 350.09	$1 999 618.96	141.68	46.61	$42 901.75	−1.46	112 018.48
ThyroidPrint	$1 964 679.15	$685 948.02	140.60	45.53	$15 066.82	−2.54	453 064.30
ThyroSPEC	$1 714 278.06	$435 546.93	134.68	39.61	$10 995.78	−8.46	165 664.26
ThyGeNEXT/ThyraMIR	$2 257 083.15	$978 352.02	141.76	46.69	$20 955.52	−1.38	622 103.64
Mir-THYpe	$2 200 033.20	$921 302.07	139.97	44.89	$20 521.71	−3.17	288 463.53
Reference Standard	$3 115 130.97	$1 836 399.84	143.14	48.07	$38 205.53	0.00	N/A
Current Care	$1 278 731.13	N/A	95.07	N/A	N/A	48.51	38 205.53

Abbreviations: MT, molecular test; ICERs, incremental cost-effectiveness ratios; QALY, quality-adjusted life year.

The molecular test with the highest surgery avoidance rate was ThyGeNEXT/ThyraMIR at 70.9%, and the test with the best diagnostic accuracy was ThyGeNEXT/ThyraMIR and 90.8% (Table 6).

**Table 6.** Cost-Minimization Analysis, Surgery Avoidance Rates, and Test Diagnostic Accuracy.

Molecular test	Cost per year ($CAD)	Compared to Reference Standard model	Compared to Current Care model	Surgery avoidance rate	Diagnostic accuracy
Afirma	$3 196 107.14	$80 976.17	$1 917 376.01	53.5%	72.9%
ThyroSeqV3	$3 070 302.53	($44 828.44)	$1 791 571.40	64.6%	84.5%
ThyroidPrint	$1 756 631.59	($1 358 499.38)	$477 900.46	69.2%	88.6%
ThyroSPEC	$1 610 254.28	($1 504 876.69)	$331 523.15	53.5%	68.5%
ThyGeNEXT/ThyraMIR	$2 026 744.78	($1 088 386.19)	$748 013.65	70.9%	91.0%
Mir-THYpe	$2 014 276.45	($1 100 854.52)	$735 545.32	64.7%	83.5%

Four out of 6 Molecular Testing strategies led to decreased number of surgeries compared to Current Care. The exception being the Afirma and ThyroSeqV3 strategy where 184 and 166 patients, respectively, tested positive and would undergo diagnostic lobectomy compared to the 149 who underwent lobectomy in Current Care.

## Discussion

This study evaluated the cost-effectiveness of molecular testing for indeterminate thyroid nodules (Bethesda III and IV) within a Manitoba context. The indeterminate nodule resection rate of 36% is comparable to other sites.^4,6^ The surgical costs vary among provinces, with Manitoba on average ending at $7082.61 per person. Although surgical costs are relatively low, the larger issue of wait times remains.

The cost analysis suggests that reflexive thyroid molecular testing with ThyroidPrint, ThyroSPEC, ThyGeNEXT/ThyraMir, Afirma, ThyroSeqv3, and mir-THYpe molecular tests is more costly per year than Current Care but less costly than the Reference Standard. The ICER for all tests except Afirma were below the WTP threshold of $50 000 per QALY making the addition of those molecular tests cost-effective compared to Current Care.

The addition of thyroid molecular testing to the treatment algorithm has a slight upfront cost associated, anywhere from $800 to $4785 per person. The cost-effectiveness depends on QALY in each circumstance (lobectomy vs surveillance), age at diagnosis, risk of malignant transformation, and cost of test. It is also sensitive to changes in QALY utility. A large portion of costs within the analysis resulted from the cost of surgery (Supplemental Materials). This study did not address Bethesda III or IV cytopathology separately. A cost-effectiveness sub-analysis was performed at various malignancy prevalences, which did not show change molecular testing cost-effectiveness, except for Afirma. Future directions include separate modeling for each risk category to improve accuracy.

Within our institution, approximately half of patients remain overnight in hospital due to various perioperative concerns such as obstructive sleep apnea, incurring costs for admission and monitoring. The surgical costs are generally fixed; therefore, strategies aimed at avoiding surgery become cost-effective. The true benefit of thyroid molecular testing lies in increasing the accuracy of diagnosis. Although avoidance of unnecessary surgeries has been noted since the commencement of molecular testing, recent evidence from Huang et al notes that in the United States, confounding factors exist, including the publication of the ATA 2015 guidelines and overall decrease in aggressive investigations of thyroid nodules.^22^

The results in the literature are not consistent. A previously conducted Canadian cost analysis show cost-effectiveness with molecular testing. In 2021, The Institut National d’Excellence en Santé et en Services Sociaux in Quebec evaluated both ThyroSeq and Afirma, comparing the cost of molecular testing to diagnostic lobectomy. Due to the comparatively higher cost of diagnostic lobectomy in Quebec,^7^ molecular testing scenarios were found to be less costly. In a follow-up cost-benefit analysis comparing diagnostic surgery to molecular testing over a 3-year period, having a benign rate of 72.6% was found to save $0.61 million per year in a Canadian public healthcare system.^21^ However, the comparative scenarios assumed universal hemithyroidectomy and are not representative of clinical practice where some patients would opt to observe.

The Ontario Health Technology Assessment found that the addition of molecular testing would increase total costs by $2313 to $2664 per patient. Such variances may be attributed to the differences in molecular test pricing used in this study (Afirma and ThyroSeqv3 at $4785) as well as decreased cost of surgery compared to Manitoba.^6^ A similar study in 2022 from the University of Calgary evaluated the cost-effectiveness of the Afirma test ($4938) versus diagnostic lobectomy ($4937) in Alberta.^20^ They concluded a cost-effective threshold to avoid surgery was $5000. They also concluded that a cost-neutral threshold was $2778 or less. As a result, Alberta has locally produced a molecular test, ThyroSPEC, that is, less than the cost-neutral threshold and have implemented routine molecular testing. Most recently, MacKay et al conducted a cost-effectiveness analysis of molecular testing for indeterminate thyroid nodules in Nova Scotia using similar methodology. They reported a mean cost per patient of $6431 with implementation of molecular testing and a potential effectiveness of 64% in avoiding unnecessary surgeries. Additionally, their analysis demonstrated an increase in hours of surgical availability, highlighting the broader healthcare system benefits of molecular testing adoption.

Our findings in Manitoba are consistent with these results, further supporting the cost-effectiveness of molecular testing across different Canadian provincial contexts. There is significant variation in cost estimates that exists in other provinces which may account for the conflicting evidence.

The primary objective of this study was to compare molecular testing strategies as a group against Current Care, rather than to identify the single most cost-effective molecular test. Selection of a specific molecular test may vary across institutions based on local practice patterns, malignancy prevalence, surgical costs, test pricing, operational constraints, and healthcare system priorities. Afirma and ThyroSeqv3 are the most used molecular tests in cost analyses; however, other molecular tests have since been developed, such as ThyroidPrint and mir-THYpe, and have become less costly with comparable NPV, PPV, sensitivity, and specificities.

The costliest molecular testing strategy with QALY included was Afirma, followed by ThyroSeqV3, mir-THYpe, ThyGeNEXT/ThyraMir, ThyroidPrint, and ThyroSPEC. In the ThyGeNEXT/ThyraMir strategy, the number of false negatives was the lowest at 4 people and is one of the tests with the highest NPV (97%). ThyroidPrint was one of the least costly of the tests, with a false-negative result of 8 people.

This study is strengthened by its use of real-world, population-based data reflecting the actual management practices and surgical rates in Manitoba, providing generalizability to a Canadian single-payer healthcare setting. While prior analyses have assumed universal surgical management for indeterminate nodules, this study realistically modeled shared decision-making and variable management strategies. By comparing multiple molecular testing platforms rather than focusing solely on Afirma or ThyroSeqV3, the study provides a modern evaluation of novel cost-effective tests such as ThyroidPrint and ThyroSPEC, reflecting the evolving diagnostic landscape. By accounting for QALYs, this study delivers a policy-relevant assessment of the clinical and economic impact of molecular testing in a resource-constrained environment.

### Limitations

This study has several limitations. First, the model assumes that nodules managed with surveillance remain stable and are discharged without further follow-up. This excludes nodules that grow and trigger repeat FNAB based on ATA guidelines outside the 1 year model timeframe.^5^

The binary interpretation of molecular test results is a simplification of clinical practice, whereas in practice they represent a spectrum of risk.

This likely overestimates surgical rates in the Molecular Testing arm and may result in conservative estimates of cost.

Similarly, the assumption that all patients with negative molecular tests avoid surgery does not reflect clinical practice, where 6% to 13% of patients with benign molecular results elect for surgery due to symptoms, preference, or other clinical factors.^6^ This bidirectional deviation was not modeled but may partially offset each other.

The model did not initially stratify Bethesda III and IV nodules separately, although both risk categories share variable and overlapping malignancy rates.^5^ Limited availability of Bethesda-specific outcomes for molecular test may reduce accuracy in malignancy risk estimation and cost-effectiveness results.

Additionally, the absence of TI-RADS within the model limits malignancy risk estimation and may affect downstream management decisions.

The 1 year time horizon, while sufficient to capture the immediate diagnostic pathway, does not account for the long-term surveillance costs that would accrue over the 5-year period recommended by TI-RADS guidelines. This may underestimate the true cost of the Current Care pathway for patients who remain under surveillance.

Completion thyroidectomy for malignant pathology was not included, as the scope of this study was intentionally limited to the initial diagnostic phase and is beyond the diagnostic cost-effectiveness question addressed in this analysis.

The QALY calculations were driven primarily by the single mortality and estimated false-negative risks. Other factors that may influence quality of life, such as anxiety, surgical complications, and the psychological impact of a cancer diagnosis, were not directly measured but were incorporated through published utility estimates.

Finally, although real-world costing enhances internal validity, differences in malignancy prevalence, cytopathology practices, and surgical resource availability across institutions may limit applicability outside of Manitoba. Future studies incorporating the full treatment pathway, including completion thyroidectomy rates and associated complications such as hypoparathyroidism, long-term surveillance outcomes, cancer progression risks, and patient-reported outcomes would provide a more comprehensive economic evaluation.

## Conclusion

This study suggests that the addition of thyroid molecular testing is cost-effective in most circumstances but especially effective compared to the Reference Standard of diagnostic lobectomy. Furthermore, in a resource restricted environment, molecular testing will improve the allocation of resources by prioritizing patients more likely to have malignancy while resulting in cost savings to the health system. Surveillance alone is ineffective in preventing aggressive transformation of differentiated thyroid cancers. This study contributes to the body of evidence suggesting molecular testing is cost-effective and has potential to influence political decision-making in health care spending.

## Supplemental Material

sj-docx-1-ohn-10.1177_19160216261479282 – Supplemental material for Cost-Effectiveness of Thyroid Nodule Molecular Testing in Manitoba Supplemental material, sj-docx-1-ohn-10.1177_19160216261479282 for Cost-Effectiveness of Thyroid Nodule Molecular Testing in Manitoba by Erika Lee, Rana Chakrabarti, Norbert Viallet, Alok Pathak, Suhail Sayed, Paul Kerr and Ciaran Lane in Journal of Otolaryngology - Head & Neck Surgery

## Data Availability

Data from this project will be available from the corresponding author upon request.
