# Supplementary material for: Cost-Effectiveness of Thyroid Nodule Molecular Testing in Manitoba
Source: J Otolaryngol Head Neck Surg. 2026 Sep 18;55:19160216261479282. doi: 10.1177/19160216261479282 (PMC13724927; doi:10.1177/19160216261479282)
Supplement: Supplementary file 1 [file sj-docx-1-ohn-10.1177_19160216261479282.docx]

Supplemental material

Table 1: Current Care pathway micro-costing

|  |  | Components | Breakdown | Cost ($CAD) | Subtotal per case | Total 2023 |
| --- | --- | --- | --- | --- | --- | --- |
| **Current care** | **Lobectomy (149)** | Surgery | 2 hours operative time 1 | $3,000.00 |  |  |
|  |  |  | Surgery unit fees 2 | $2,710.00 |  |  |
|  |  |  | Surgeon fee 3 | $747.61 |  |  |
|  |  |  | Assistant wage 3 | $75.00 |  |  |
|  |  |  | Lobectomy pathology 4 | $530.00 |  |  |
|  |  |  | Complications 5 | $20.00 |  |  |
|  |  | Follow up | 1 Thyroid ultrasound 3,6 | $139.24 |  |  |
|  |  |  | 2 Follow up visits ($112.42 x 2) 3 | $224.84 |  |  |
|  |  |  | 2 Thyroid bloodwork 7 | $96.00 |  |  |
|  |  |  |  | Total surgical cost/patient | $7,542.69 | $1,123,860.81 |
|  | **Observation surveillance (264)** | Ultrasound guided biopsy | 1 Ultrasound guided FNA 3,6 | $324.21 |  |  |
|  |  |  | 1 FNA cytology 4 | $150.00 |  |  |
|  |  |  | Follow up visit ($112.42) 3 | $112.42 |  |  |
|  |  |  |  | Total surveillance cost/patient | $586.63 | $154,870.32 |
|  |  |  |  | **Total Current care** | $8,129.32 | $1,278,731.13 |
|  | **QALY lost** | utility 0 | 1 preventable death (age 33) |  |  |  |
|  |  | Life years (up to 82 years) | 82-33 = 49 QALY |  |  |  |
|  |  | $50,000 per QALY |  |  |  |  |

1 - Email communication with HSC provincial surgery lead

2- Based on day surgery and short stay surgery, Admitting HSC department

3- Manitoba physicians Manual billing guide, office visit 8540, 8447 ($112.42), surgeon's fee 4912 ($747.61), radiology consultation 8550 ($86.05), radiology biopsy 6191 ($98.92), ultrasound physician interpretation 7302 ($39.24)

4- Email communication with lead for pathology services

5 - Post lobectomy complications based on literature estimating risk of post operative hematoma (1.5%) or infection (1%) leading to ER visit ($90.02 8540 + $250 estimated for labs and nursing), plus 1% risk of laryngeal nerve injury leading to specialist visit ($90.02)

6- Email communication Shared health clinical change lead diagnostic services: facility fee ($100)

7- Email communication Shared Health biochemistry lab services

Table 2: Molecular Testing pathway micro-costing

|  |  | **Components** | **Breakdown** | **Cost ($CAD)** | **Subtotal per case** |
| --- | --- | --- | --- | --- | --- |
| **Molecular testing** | **Molecular test** |  |  |  |  |
|  | **Positive (Lobectomy)** | Surgery | 2 hours operative time 1 | $3,000.00 |  |
|  |  |  | Surgery unit fees 2 | $2,710.00 |  |
|  |  |  | Surgeon fee 3 | $747.61 |  |
|  |  |  | Assistant hourly wage 3 | $75.00 |  |
|  |  |  | Lobectomy pathology 4 | $530.00 |  |
|  |  |  | Complications 5 | $20.00 |  |
|  |  | Follow up | 1 Thyroid ultrasound 6 | $139.24 |  |
|  |  |  | 2 Follow up visits* ($112.42 x 2) 3 | $224.84 |  |
|  |  |  | 2 Thyroid bloodwork 7 | $96.00 |  |
|  |  |  |  | Subtotal positive test/person | $7,542.69 |
|  | **Negative** | No surveillance | 1 Follow up visit* ($112.42 x 1) 3 | $112.42 |  |
|  |  |  |  | Subtotal negative test/person | $112.42 |

1 - Email communication with HSC provincial surgery lead

2- Based on day surgery and short stay surgery, Admitting HSC department

3- Manitoba physicians Manual billing guide, office visit 8540, 8447 ($112.42), surgeon's fee 4912 ($757.61), radiology consultation 8550 ($86.05), radiology biopsy 6191 ($98.92), ultrasound physician interpretation 7302 ($39.24)

4- Email communication with lead for pathology services

5 - Post lobectomy complications based on literature estimating risk of post operative hematoma (1.5%) or infection (1%) leading to ER visit ($90.02 8540 + $250 estimated for labs and nursing), plus 1% risk of laryngeal nerve injury leading to specialist visit ($90.02)

6- Email communication Shared health clinical change lead diagnostic services: facility fee ($100)

7- Email communication Shared Health biochemistry lab services

Table 3: Reference Standard pathway micro-costing

|  |  |  | **Components** | **Breakdown** | **Cost ($CAD)** | **Subtotal per case** | **Total 2023** |  |
| --- | --- | --- | --- | --- | --- | --- | --- | --- |
|  | **Reference Standard** | **Lobectomy (413)** | Surgery | 2 hours operative time 1 | $3,000.00 |  |  |  |
|  |  |  |  | Surgery unit fees 2 | $2,710.00 |  |  |  |
|  |  |  |  | Surgeon fee 3 | $747.61 |  |  |  |
|  |  |  |  | Assistant wage 3 | $75.00 |  |  |  |
|  |  |  |  | Lobectomy pathology 4 | $530.00 |  |  |  |
|  |  |  |  | Complications 5 | $20.00 |  |  |  |
|  |  |  | Follow up | 1 Thyroid ultrasound 6 | $139.24 |  |  |  |
|  |  |  |  | 2 Follow up visits ($112.42 x 2) 3 | $224.84 |  |  |  |
|  |  |  |  | 2 Thyroid bloodwork 7 | $96.00 |  |  |  |
|  |  |  |  |  | **Total Reference Standard** | $7,542.69 | $3,115,130.97 |  |
| 1 - Email communication with HSC provincial surgery lead | | | | | | | | |
| 2- Based on day surgery and short stay surgery, Admitting HSC department | | | | | | | | |
| 3- Manitoba physicians Manual billing guide, office visit 8540, 8447 ($112.42), surgeon's fee 4912 ($757.61), radiology consultation 8550 ($86.05), radiology biopsy 6191 ($98.92), ultrasound physician interpretation 7302 ($39.24) | | | | | | | | |
| 4- Email communication with lead for pathology services | | | | | | | | |
| 5 - Post lobectomy complications based on literature estimating risk of post operative hematoma (1.5%) or infection (1%) leading to ER visit ($90.02 8540 + $250 estimated for labs and nursing), plus 1% risk of laryngeal nerve injury leading to specialist visit ($90.02) | | | | | | | | |
| 6- Email communication Shared health clinical change lead diagnostic services: facility fee ($100) | | | | | | | | |
| 7- Email communication Shared Health biochemistry lab services | | | | | | | | |
|  |  | | | | | | | |
|  |  | | | | | | | |
|  |  | | | | | | | |
|  |  | | | | | | | |
|  |  | | | | | | | |
|  |  | | | | | | | |
|  |  | | | | | | | |

Table 4: Molecular test population estimates based on site prevalence of 21% and testing statistics in Table 1 (main figures and tables)

n=413

Disease n=88

True positive n=80

False negative n=8

No disease n=325

False positive n=104

True negative n=221

Table 4a: Afirma

Number of patients with and without the disease who test positive and negative

|  | Disease present | Disease absent | Total |
| --- | --- | --- | --- |
| Test positive | 80 | 104 | 184 |
| Test negative | 8 | 221 | 229 |
| Total | 88 | 325 | 413 |

n=413

Disease n=88

True positive n=82

False negative n=5

No disease n=325

False positive n=59

True negative n= 267

Table 4e: ThyroSeqV3

|  | Disease present | Disease absent | Total |
| --- | --- | --- | --- |
| Test positive | 82 | 59 | 141 |
| Test negative | 5 | 267 | 272 |
| Total | 88 | 325 | 413 |

n=413

Disease n=88

True positive n=80

False negative n=8

No disease n=325

False positive n=39

True negative n= 286

Table 4b: ThyroidPrint

|  | Disease present | Disease absent | Total |
| --- | --- | --- | --- |
| Test positive | 80 | 39 | 119 |
| Test negative | 8 | 286 | 294 |
| Total | 88 | 325 | 413 |

n=413

Disease n=88

True positive n=62

False negative n=26

No disease n=325

False positive n=104

True negative n= 221

Table 4c: ThyroSPEC

|  | Disease present | Disease absent | Total |
| --- | --- | --- | --- |
| Test positive | 62 | 104 | 166 |
| Test negative | 26 | 222 | 247 |
| Total | 88 | 325 | 413 |

n=413

Disease n=88

True positive n=83

False negative n=4

No disease n=325

False positive n=33

True negative n= 293

Table 4d: ThyGeNEXT/ThyraMIR

|  | Disease present | Disease absent | Total |
| --- | --- | --- | --- |
| Test positive | 83 | 33 | 116 |
| Test negative | 4 | 293 | 297 |
| Total | 88 | 325 | 413 |

Table 4f: Mir-THYpe

n=413

Disease n=88

True positive n=78

False negative n=10

No disease n=325

False positive n=59

True negative n= 267

|  | Disease present | Disease absent | Total |
| --- | --- | --- | --- |
| Test positive | 78 | 59 | 137 |
| Test negative | 10 | 267 | 276 |
| Total | 88 | 325 | 413 |

Table 5: Recalculation of ICER with Current Care QALY calculation at -40% surgical costs

|  |  | **Current Care** | | | | **Reference Standard** | |  |
| --- | --- | --- | --- | --- | --- | --- | --- | --- |
| **Molecular test** | **Cost per year** | **Difference in Cost Compared with Current Care** | **Net QALY (QALY gained - QALY lost)** | **Difference in QALY compared to Current Care** | **ICER Compared to Current Care ($/QALY)** | **Difference QALY compared to Reference Standard** | **ICER MT Compared to Reference Standard** |  |
|  |  |  |  |  |  |  |  |  |
| Afirma | $2,674,827.04 | $1,818,219.47 | 127.71 | 32.64 | $55,711.71 | -15.43 | 28,535.35 |  |
| ThyroSeqV3 | $ 2,670,843.33 | $1,814,235.75 | 141.20 | 46.13 | $39,330.22 | -1.94 | 229,244.82 |  |
| ThyroidPrint | $1,419,499.35 | $562,891.78 | 139.54 | 44.47 | $12,658.90 | -3.60 | 470,976.87 |  |
|  |  |  |  |  |  |  |  |  |
| ThyroSPEC | $1,139,968.98 | $283,361.40 | 128.93 | 33.86 | $8,368.65 | -14.21 | 139,032.63 |  |
|  |  |  |  |  |  |  |  |  |
| ThyGeNEXT/ThyraMIR | $1,698,111.68 | $841,504.10 | 140.71 | 45.64 | $18,439.19 | -2.43 | 583,223.32 |  |
|  |  |  |  |  |  |  |  |  |
|  |  |  |  |  |  |  |  |  |
|  |  |  |  |  |  |  |  |  |
| Mir-THYpe | $ 1,626,149.42 | $769,541.85 | 138.32 | 43.24 | $17,795.99 | -4.82 | 308,667.43 |  |
|  |  |  |  |  |  |  |  |  |
| Reference Standard | $3,115,130.97 | $2,258,523.40 | 143.14 | 48.07 | $46,987.63 | 0.00 | N/A |  |
|  |  |  |  |  |  |  |  |  |
| Current Care | $856,607.57 | N/A | 95.07 | N/A | N/A | 48.51 | 46,987.63 |  |
|  |  |  |  |  |  |  |  |  |

Table 6: Recalculation of ICER with Current Care QALY calculation at +40% surgical costs

|  |  | **Current Care** | | | | **Reference Standard** | |  |
| --- | --- | --- | --- | --- | --- | --- | --- | --- |
| **Molecular test** | **Cost per year** | **Difference in Cost Compared with Current Care** | **Net QALY (QALY gained - QALY lost)** | **Difference in QALY compared to Current Care** | **ICER Compared to Current Care ($/QALY)** | **Difference QALY compared to Reference Standard** | **ICER MT Compared to Reference Standard** |  |
|  |  |  |  |  |  |  |  |  |
| Afirma | $3,717,387.24 | $2,016,532.55 | 127.71 | 32.64 | $61,788.18 | -15.43 | 36,797.56 |  |
| ThyroSeqV3 | $ 3,469,761.73 | $1,768,907.05 | 141.20 | 46.13 | $38,347.55 | -1.94 | 420,740.91 |  |
| ThyroidPrint | $2,093,763.83 | $392,909.14 | 139.54 | 44.47 | $8,836.15 | -3.60 | 608,684.96 |  |
|  |  |  |  |  |  |  |  |  |
| ThyroSPEC | $2,080,539.58 | $379,684.90 | 128.93 | 33.86 | $11,213.42 | -14.21 | 155,185.60 |  |
|  |  |  |  |  |  |  |  |  |
| ThyGeNEXT/ThyraMIR | $2,355,377.88 | $654,523.20 | 140.71 | 45.64 | $14,342.03 | -2.43 | 794,276.07 |  |
|  |  |  |  |  |  |  |  |  |
|  |  |  |  |  |  |  |  |  |
| Mir-THYpe | $ 2,402,403.48 | $701,548.79 | 138.32 | 43.24 | $16,223.62 | -4.82 | 390,301.15 |  |
|  |  |  |  |  |  |  |  |  |
| Reference Standard | $4,285,178.14 | $2,584,323.46 | 143.14 | 48.07 | $53,765.76 | 0.00 | N/A |  |
|  |  |  |  |  |  |  |  |  |
| Current Care | $1,700,854.69 | N/A | 95.07 | N/A | N/A | 48.51 | 53,765.76 |  |
|  |  |  |  |  |  |  |  |  |
